# eQTLMAPT: Fast and Accurate eQTL Mediation Analysis With Efficient Permutation Testing Approaches

**DOI:** 10.3389/fgene.2019.01309

**Published:** 2020-01-09

**Authors:** Tao Wang, Qidi Peng, Bo Liu, Xiaoli Liu, Yongzhuang Liu, Jiajie Peng, Yadong Wang

**Affiliations:** ^1^ School of Computer Science and Technology, Harbin Institute of Technology, Harbin, China; ^2^ Department of Neurology, Zhejiang Hospital, Hangzhou, China; ^3^ School of Computer Science, Northwestern Polytechnical University, Xi’an, China

**Keywords:** trans-eQTL, cis-eQTL, mediation analysis, multiple testing control, permutation test, gene regulation

## Abstract

Expression quantitative trait locus (eQTL) analyses are critical in understanding the complex functional regulatory natures of genetic variation and have been widely used in the interpretation of disease-associated variants identified by genome-wide association studies (GWAS). Emerging evidence has shown that *trans*-eQTL effects on remote gene expression could be mediated by local transcripts, which is known as the mediation effects. To discover the genome-wide eQTL mediation effects combing genomic and transcriptomic profiles, it is necessary to develop novel computational methods to rapidly scan large number of candidate associations while controlling for multiple testing appropriately. Here, we present eQTLMAPT, an R package aiming to perform eQTL mediation analysis with implementation of efficient permutation procedures in multiple testing correction. eQTLMAPT is advantageous in threefold. First, it accelerates mediation analysis by effectively pruning the permutation process through adaptive permutation scheme. Second, it can efficiently and accurately estimate the significance level of mediation effects by modeling the null distribution with generalized Pareto distribution (GPD) trained from a few permutation statistics. Third, eQTLMAPT provides flexible interfaces for users to combine various permutation schemes with different confounding adjustment methods. Experiments on real eQTL dataset demonstrate that eQTLMAPT provides higher resolution of estimated significance of mediation effects and is an order of magnitude faster than compared methods with similar accuracy.

## Introduction

Understanding the complex functional natures of genome variants has been the focus of many studies in recent years, which provides us with advanced insights into phenotype variability and disease susceptibility (Cheng et al., 2017; Watanabe et al., 2017; Gallagher and Chen-Plotkin, 2018). Vast genome variants relevant to disease risks and other traits have been unequivocally identified by genome-wide association studies (GWAS) (Visscher et al., 2017). However, most of those traits-associated variants localize in non-coding regions, intergenic, or intronic regions, indicating that genomic variants are likely to be involved in gene regulation instead of exerting their effects through altering the protein sequence directly (Gallagher and Chen-Plotkin, 2018). To understand the complex regulatory natures of genomic variants, one of the fundamental tasks is to discover target genes which can be regulated by variants in the cell. The expression quantitative trait loci (eQTL) analysis has been proven a powerful tool in achieving this goal.

An eQTL is essentially a variant at a specific genome location with its genetic variance associates with gene expression variation in a population. Most eQTL mapping studies access the eQTL effects through association tests between the genotypes of a variant and expression profiles of a gene using regression models (Shabalin, 2012; Ongen et al., 2015). And eQTL summary statistics have been widely used in the interpretation of GWAS results and Mendelian randomization studies (Cheng et al., 2018b; Peng et al., 2019a). eQTLs can exert their regulatory effects on local gene transcriptions (*cis*-acting) and distant gene transcriptions (*trans*-acting), defined by the physical distance between an eQTL and a gene, usually using 1 Mb as a threshold or on different chromosomes for *trans*-acting associations (Ongen et al., 2015; GTEx Consortium, 2017). *cis*-acting or *trans*-acting may reflect different underlying regulation natures. For example, *cis*-eQTLs usually reside close to transcription starting sites (TSS) and might affect the gene expression directly through affecting transcription factor (TF) binding process (Nica and Dermitzakis, 2013). However, very little knowledge is known for *trans*-eQTLs due to multifaceted reasons. First, *trans*-acting effects are usually weaker than *cis*-acting, which requires a large sample size to detect the weak signals (Yao et al., 2017). Second, the number of *trans*-eQTL associations is an order of magnitude more than *cis*-eQTL associations, which brings heavy computational burdens. Third, the multiple testing problem in identifying *trans*-eQTLs results in stringent significance thresholds. And *trans*-eQTLs have been proven less replicable across studies (Innocenti et al., 2011). Therefore, most eQTL studies only focus on *cis*-eQTLs, and the mechanisms underling the regulatory effects of genetic variation on the expression of distant genes and genes in other chromosomes are largely unknown (Bryois et al., 2014).

Recent studies have shown that *trans*-eQTLs are likely involved in indirect regulations, where the *trans*-eGene can be mediated by the *cis*-eGene, which is known as the mediation effect (Pierce et al., 2014; Brynedal et al., 2017; Yang et al., 2017; Yao et al., 2017). These studies provide evidence of a *cis*-mediated mechanism that explains distal regulation of *trans*-eGenes by *trans*-eQTLs (Yao et al., 2017). Characterizing these regulatory relationships will allow us to better understand regulatory networks and the biological mechanisms underlying *trans*-eQTLs (Westra et al., 2013). To discover the mediation effect among *cis*-/*trans*-eQTL (L), *cis*-eGene (C) and *trans*-eGene (T), represented by a trio (*L*→*C*→*T*), a recently proposed work which aims to test the significance of the effect of *cis*-eGene on *trans*-eGene controlled by the genotype of *L* and confounders (Yang et al., 2017). Mathematically, by using a linear regression model, with the formula *T* = *a* + β_1_ *C* + β_2_ *G* + ΓCov + *ϵ*, where *G* represents the genotype of *L* (see details in *Material and Methods*), the objective is to test the significance of *β*_1_. In practice, this requires performing a large amount of association tests in order to scan all possible candidate trios due to related variants in linkage disequilibrium (LD). Thus, it will result in a large number of nominal statistics, i.e., *P* values, and multiple testing has to be considered in order to control the false discovery rate. A traditional solution is to use Bonferroni correction method, which multiplies the nominal *P* value with the total number of tests to get an adjusted *P* value. However, the Bonferroni method has been proven overly stringent in genomic area due to the fact that a large number of tests are not independent because of variants in LD, and this method will result in a lot of false negatives (Ongen et al., 2015).

To solve this problem, a commonly adopted strategy is to use the non-parametric permutation testing approach. The permutation test can be performed by the following steps: first, perform thousands of permutations on gene expression profiles by randomly exchanging sample IDs. Notably, to break the potential mediation effects from *C* to *T* while keeping the *cis*-eQTL and *trans*-eQTL associations, the sample ID rearrangement need to be performed within each genotype group (i.e., AA, AB, or BB) (Yang et al., 2017). Second, calculate a list of permutation statistics, under the null hypothesis of no association, by performing associations using genotypes and permuted expressions. Third, compare the nominal statistics with the distribution of permutation statistics to assess how likely the observed nominal association statistics originates from the null distribution. The permutation tests have been applied to multiple bioinformatics applications to control for multiple testing, for example, eQTL mapping (Ongen et al., 2015), allelic association analysis (Zhao et al., 2000), and biological network analyses (Wang et al., 2019). In the context of detecting mediation effect of *cis*-eGenes on *trans*-eGenes, a recently proposed algorithm named GMAC adopts permutation strategy to control for multiple testing (Yang et al., 2017). However, it suffers from a main drawback: it relies on performing a fixed number, usually thousands of permutations per trio, to balance the running time and *P* value resolution empirically estimated. For example, 10,000 permutations can derive *P* value at a resolution of 10^−4^ at the best circumstance. There is no efficient built-in permutation scheme, which makes its practical application very time-consuming and not accurate in estimating significance of mediation effects.

In this work, we present eQTLMAPT, an R package which improves upon GMAC (Yang et al., 2017) by implementing faster and more efficient permutation-based multiple testing correction approaches. Besides the traditional fixed permutation scheme, eQTLMAPT also provides 1) the adaptive permutation scheme which prunes the permutation process opportunely; 2) the approximation of the tail of null distribution using generalized Pareto distribution (GPD) model, which allows the user to accurately estimate adjusted *P* values at any significance level in a short running time; and 3) flexible choices of different confounding factors adjustment methods. In addition, eQTLMAPT provides flexible interfaces for users to combine different features and perform the proper permutation scheme based on their practical needs. Experiments on a real eQTL dataset demonstrate that eQTLMAPT is an order of magnitude faster than GMAC, and its estimated significance has a much higher resolution than the compared method.

## Material and Methods

### Overview

To efficiently identify *cis*-eGene mediators of *trans*-eQTLs in whole genome, we developed eQTLMAPT, an R package to perform mediation analysis with multiple permutation schemes and flexible covariate adjustment strategies. The core regression models we used in mediation analysis is similar to the model used in the recently proposed method, GMAC (Yang et al., 2017). The models can be formalized as Equations 1, 2, and 3, where *G* represents the genotype of single nucleotide polymorphism (SNP)*L*; *C*, and *T* represent gene expression levels of *cis*-eGene and *trans*-eGene, respectively; Cov represents covariates; and *ϵ* represents the error term following normal distribution. For the trio (*L*,*C*,*T*), we assume *L* is significantly associated with *C* and *T* by testing *β*_1_ ≠ 0 and β_2_ ≠ 0 in the linear models, with *β* estimated by least-squares fitting. The statistic of mediation analysis here is to test the mediation effect of *cis*-eGene *C* on *trans*-eGene *T* while controlling for the effects of eQTL *L*, covariants Cov. The null hypothesis is *H*_0_:*β*_3_ = 0.

(1) $$C=a_{1}+\beta_{1}G+\Gamma_{1}Cov+\varepsilon_{1}$$

(2) $$T=a_{2}+\beta_{2}G+\Gamma_{2}Cov+\varepsilon_{2}$$

(3) $$T=a_{3}+\beta_{3}C+\beta_{4}G+\Gamma_{3}Cov+\varepsilon_{3}$$

Our method can be separated into two main steps: first, we calculate the nominal association statistic, *z* = *β*_3_/*se*, in Equation 3, where *se* represents the standard error of *β*_3_. Second, to account for multiple testing in assessing the significance of the mediation effect, we perform within-genotype group permutations of *cis*-eGene transcripts *C* to empirically characterize the null distribution of mediation effects (i.e., the distribution of *z* scores expected under the null hypothesis of no mediation effect, denoted by vector *Z*_0_). The purpose of within-genotype group permutation is to break the potential mediation effects from *C* to *T* within each genotype group (i.e., AA, AB, or BB) while keeping the *cis*-eQTL and *trans*-eQTL associations. The adjusted empirical *P* value of mediation test would finally be calculated by comparing the observed mediation statistic *z* with the permutation statistics *Z*_0_ under the null.

To obtain the null distribution of mediation effects, i.e., *Z*_0_, and provide users with flexible choices, we implemented three permutation schemes in our package: 1) fixed permutation scheme, which generates *N* permutation datasets (*Estimation of P Values Under Fixed Permutation Scheme*); 2) adaptive permutation scheme, which prunes the permutation process when there are too many null statistics better than the observed *z* statistic (*Calculate Empirical P Value Using Adaptive Permutation Scheme*); and 3) GPD approximation, which models the tail of the null distribution *via* a drastically reduced number of null statistics and estimates *P* value with higher resolution (*Model the Tail of the Null Distribution Using GPD*). To deal with complex hidden confounding effects, we also adopt an adaptive confounder adjustment method (Yang et al., 2017) and a fixed confounder adjustment method incorporating the three permutation schemes (*Confounding Factors Adjustment*).

### Estimation of *P* Values Under Fixed Permutation Scheme

The associations of trios (*L*,*C*,*T*) we aim to test are not independent due to the fact that multiple SNPs are correlated because of LD. Traditional multiple testing correction methods like Bonferroni and Benjamini–Hochberg correction, which give a global significance threshold based on all nominal *P* values, prove to be overly stringent and may result in false negatives in such correlated genomic analyses. Thus, we adapt permutation-based testing approaches to assess the significance in association test for each trio (*L*, *C*, *T*) (Equation 3). Permutation test is a widely used non-parametric method in many bioinformatics applications. It generates a null statistic distribution by random permutations and then assesses how likely the observed statistic obtained in the nominal association originates from the null distribution.

Assume the nominal mediation statistic *z* = *β*_3_/*se* is assessed for a trio (*L*, *C*, *T*) by Equation 3, where *se* is the standard error of *β*_3_. Given a fixed number of *N*, we perform *N* times permutations within-genotype groups for *cis*-eGene *C* by randomly permuting sample labels in each genotype group, i.e., AA, AB, and BB. It will generate *N* null mediation statistics, denoted by $Z_{0}=\{z_{0}^{1},z_{0}^{2},\ldots ,z_{0}^{N}\}$, where $z_{0}^{i}$ is in absolute value, *i∈*[1,*N*]. If *M* null statistics in *Z*_0_ are stronger than the observed statistic |*z*|, the empirical *P* value is assessed by Equation 4, where pseudo-count 1 is added to avoid meaningless denominator.

(4) $$P_{\mathrm{fixed}}=\frac{M+1}{N+1}$$

The strategy of fixed permutation scheme is direct, easy to implement, and adopted by most permutation testing approaches. However, the adjusted *P* value has lower bound limitation that $P_{fixed}\geq \frac{1}{N+1}$. That means we have to increase the fixed number of *N* to get precise *P* value estimates for strong mediation effects with smaller *P* values, which will tremendously increase the computational costs. For example, if the true *P* value is 10^−6^ for a trio, at least 1 million permutations should be performed to achieve the precise *P* value. But for most trios, with true *P* values larger than 10^−3^, 1 million permutations would be a waste of resources because thousands of permutations could lead to precise *P* values. To solve this problem, we implemented an adaptive permutation strategy in eQTLMAPT to prune permutations once we observe too many null statistics stronger than the nominal statistic *z* of mediation analysis.

### Calculate Empirical *P* Value Using Adaptive Permutation Scheme

The basic idea of adaptive permutation strategy is to perform more permutations for significant trios while decreasing the number of permutations for insignificant trios. This is because insignificant trios could be assessed with fewer permutations than significant ones. By setting a significance level, *α* = 0.05 for example, and a maximum permutation times *N*, in case of indefinitely running the process, we define the pruning threshold *K* = α*N, and usually *K* << *N*. For each trio (*L*,*C*,*T*), if we observe more than or equal to *K* null statistics that $\left|z_{0}^{i}|>|z\right|$ or we reach the maximum permutation upper bound *N*, the permutations process will be stopped. Suppose Γ times of permutations are executed in total and *M* null statistics are found to be stronger than the observed statistic |*z*|, the adjusted *P* value is given by Equation 5.

(5) $$P_{adaptive}=\frac{\min\left(K+1,M+1\right)}{\min\left(\Gamma +1,N+1\right)}$$

For example, given *N* = 10,000 and *α* = 0.05, then *K* = 500, and assume we have performed 800 times of permutation for a trio and find *K* null statistics stronger than nominal statistic *z*. Then, we stop performing further permutations and the final adjusted *P* value = 501/801. In this case, only 800 times permutations are needed instead of 10,000 times in the fixed permutation scheme. This strategy tremendously reduces the number of permutations required for insignificant trios; however, the lower bound of adjusted *P* value still exists, which is 1/(*N* + 1). To solve the lower bound problem, we approximate the tail of null statistics distribution by generalized Pareto distribution and estimate the small *P* values at any significance level without the limitation of lower bound.

### Model the Tail of the Null Distribution Using GPD

It is critical to accurately estimate small *P* values especially in large-scale genomic analyses, where huge numbers of associations are simultaneously tested. To determine precise small *P* values at any significance level without performing all possible permutations, we implemented a *P* value approximation method based on GPD, which has been widely used in modeling extreme values (Knijnenburg et al., 2009). The basic methodology is to estimate the small permutation *P* values using extreme value theory by fitting extreme permutation values originating from the tail of null distribution with generalized Pareto distribution (Gumbel, 2012). And it has been proven that the GPD approximation method can lead to precise estimation of small *P* values using much fewer permutations compared with fixed number of permutation approach (Knijnenburg et al., 2009).

In our case, given permutation statistics set $Z_{0}=\{z_{0}^{1},z_{0}^{2},\ldots ,z_{0}^{N}\}$ and nominal mediation statistic *z* of a trio (*L*,*C*,*T*), we suppose both *z* and $z_{0}^{i}\in Z_{0}$ are in absolute value, and elements in *Z*_0_ are sorted in decreasing order, i.e., $z_{0}^{i}\geq z_{0}^{j}$, *i*<*j*. Define *Nexc* as the number of exceedances (extreme values), and $Y_{0}=\{z_{0}^{1},z_{0}^{2},\ldots ,z_{0}^{N_{exc}}\}$,*Y*_0_⊂*Z*_0_, and exceedance threshold $t=\left(z_{0}^{N_{exc}}+z_{0}^{N_{exc}+1}\right)/2$, such that *z*_0_ > *t*, if *z*_0_∈*Y*_0_. Then, we calculate *z*_0_−*t* for each element *z*_0_∈*Y*_0_ to get a vector of exceedances $X_{0}=\left\{x_{0}^{1},x_{0}^{2},\text{...}x_{0}^{N_{exc}}\right\}$, where $x_{0}^{i}=z_{0}^{i}-t,x_{0}^{i}\in X_{0},z_{0}^{i}\in Y_{0}$. Next, exceedances in *X*_0_ are used to fit the tail of the null distribution modeling by GPD. The GPD has cumulative distribution function (CDF) shown in Equation 6.

(6) $$F(x)=\{\begin{array}{ll} 1–{\left(1–\frac{kx}{a}\right)}^{\frac{1}{k}}, & k\neq 0\\ 1-e^{\frac{-x}{a}}, & k=0 \end{array}$$

The *a* and *k* are scale parameter and shape parameter, respectively, and the range of *x* requires $0\leq x\leq \frac{a}{k}$ for *k* > 0, and *x* ≥ 0 for *k* ≤ 0. If *x* falls out of these ranges, the GPD estimated *P* values will be zeros, i.e., $k>0,x>\frac{a}{k}$. Maximum likelihood (ML) is used to estimate the two parameters *a* and *k* in *F*(*x*) given *X*_0_. The goodness-of-fit test of the Anderson–Darling statistic is used to evaluate whether the exceedances follow the GPD (Choulakian and Stephens, 2001). Finally, the permutation test *P* value of the GPD approximation is computed as shown in Equation 7, where *z* represents the absolute value of the nominal mediation statistic.

(7) $$P_{gpd}=\frac{N_{exc}}{N}(1-F(z-t))$$

*N*_exc_ is initialized as minimum value between 250 and number of permutation tests by default. If it fails to fit GPD (goodness-of-fit test *P* ≤ 0.05), then iteratively reduce *N*_exc_ by 10 until a good fit is achieved. Besides, the GPD approximation can only be used when the nominal mediation statistic *z* is in the range of extreme permutation null statistics (tail of null distribution). For example, if *z* is in the middle of the null distribution, this method cannot be applied. To specify, let *M* be the number of permutation values that exceed the test statistic *z*, if *M* < N*α, *α* = 0.01 in default, GPD approximation will be performed; otherwise, fixed permutation scheme will be performed. The detailed methods have been described in Knijnenburg et al. (2009), and we implemented this method with R language in our package to accurately estimate the mediation significance with much fewer permutations.

### Confounding Factors Adjustment

The presence of heterogeneous known or latent unmeasured covariates that affect genotype and phenotype (gene expression in our context) is a major source of bias in the mediation analysis, which needs to be adjusted. The common sources of covariates, such as batch effects, age, sex, postmortem interval (PMI), RNA integrity number (RIN), and population stratification, are associated with either samples or individuals. The latent unwanted covariates can be identified by methods like principal component analysis (PCA) (Abdi and Williams, 2010), surrogate variables analysis (SVA) (Leek et al., 2012), and probabilistic estimation of expression residuals (PEER) (Stegle et al., 2012).

In our package, we adopt two covariates adjustment strategies: fixed confounder adjustment strategy and adaptive confounder adjustment strategy. The first one is to directly pass the user-given PCs/SVs or PEER factors together with known covariates into the *Cov* variable in Equation 3 when performing mediation analysis. The second way is proposed in GMAC (Yang et al., 2017), which adaptively selects hidden covariates for each trio. In brief, this method first identifies a pool of hidden covariates, represented by *H*, which can be supplied by users or identified with PCA on expression profiles automatically [first 30 principal components (PCs) in default]. Then, for each trio (*L*,*C*,*T*), only a small number of PCs will be selected from *H* for adjustment based on the correlations between PCs and *C*,*T*. And experiments demonstrated that this adaptive covariates selection method improved power and precision in mediation analysis (Yang et al., 2017). Notably, both covariates adjustment strategies can be flexibly selected by users for each of the three permutation schemes introduced above.

### ROSMAP Dataset and Preprocessing

#### ROSMAP Study and Dataset

The Religious Orders Study (ROS) (A Bennett et al., 2012a) and Memory and Aging Project (MAP) (A Bennett et al., 2012b) are two longitudinal cohort studies of aging and Alzheimer’s disease (AD). We downloaded the gene expression, genotype, and clinical dataset of ROSMAP Study from Synapse platform (ID: syn3219045) with approval. RNA samples were obtained from the homogenate of the dorsolateral prefrontal cortex of 724 subjects and RNA sequencing (RNA-seq) data have been processed into read count table using standard pipeline (syn9702085) (Mostafavi et al., 2018). DNA samples were from whole blood and genotype profiles of 1,179 subjects were calculated from whole-genome sequencing (De Jager et al., 2018). Only neuropathologically healthy individuals (cogdx score ≤3, no Alzheimer’s disease and no dementia) with both genotype data and RNA-seq data passing quality controls were used in eQTL analysis, which downsized the sample size to *N* = 334.

#### Genotype Processing

We applied PLINK2 (v1.9beta) (Chang et al., 2015) and in-house scripts to perform rigorous subject and SNP quality control (QC) for genotype dataset derived from WGS. To QC in SNP level, we removed SNPs with genotype call rate <95%, with Hardy–Weinberg equilibrium testing *P* < 10^−6^, informative missingness test *P* < 10^−9^, and with minor allele frequency (MAF) < 0.05 seperately. To QC in subject level, we removed subjects with call rate <95%, with outlying heterozygosity rate based on heterozygosity *F* score (beyond 4*sd from the mean *F* score), and with gender mismatch. We also performed IBS/IBD filtering: pairwise identity-by-state probabilities were computed for removing both individuals in each pair with IBD > 0.98 and one subject of each pair with IBD > 0.1875. To test for population substructure, we performed PCA using smartPCA in ENGINSOFT (Patterson et al., 2006).

#### Gene Expression Profiles Processing

Stringent quality controls and normalization steps were also performed for gene expression profiles. Gene read count derived from RNA-seq was normalized to TPM (transcripts per kilobase million) by scaling gene length (union of exon length) and sequencing depth. We removed samples with gender mismatch by checking gender-specific expression genes XIST and RPS_4_Y_1_. Sample outliers with problematic gene expression profiles were detected and removed based on hierarchical clustering (AC’t Hoen et al., 2013). Genes with low expression were also removed by keeping genes with >0.1 TPM in at least 20% of samples and ≥6 reads in at least 20% samples. For normalization, gene expression values were quantile normalized after log10-transformed. SVA package was applied for removing batch effect and adjusting age, sex, RIN, PMI, and latent covariates. Residuals were outputted for downstream eQTL analysis.

#### eQTL Mapping and Mediation Analysis

MatrixEQTL (Shabalin, 2012) was used for *cis*/*trans*-eQTL mapping using additive linear model. In *cis*-eQTL analysis, variants (SNPs and indels) within 1 M upstream and downstream from the TSS were tested for association with gene expression traits. And variants beyond the ±1M window were associated with the gene expression traits in *trans*-acting manner. For *cis*-eQTL results, a significance level of false discovery rate (FDR) ≤0.05 was used. And for *trans*-eQTL results, we adopt a global significance level *P* < 1 × 10^−8^ because of the tremendous amount of *trans*-associations and weak *trans*-eQTL effects.

For biological discovery, mediation analyses with adaptive permutation scheme and GPD approximation (*N* = 10,000, *α* = 0.05) were applied for all candidate trios (*L*,*C*,*T*), where eQTL *L* was significantly associated with *cis*-eGene *C* (FDR ≤ 0.05; Equation 1) and *trans*-eGene *T* (*P* < 1 × 10^−8^; Equation 2). For performance comparison, mediation analyses were performed in multiple scenarios described in the *Results* section.

## Results

### Candidate (*L*, *C*, *T*) Trios Detected in ROSMAP Dataset

After stringent quality controls for both RNA-seq and genotyping data (*ROSMAP Dataset and Preprocessing*), 26,662 gene transcripts and 6,736,714 variants (including SNPs and indels) of 334 subjects were left for eQTL analysis. We detected 3,195,073 significant *cis*-eQTL associations, representing 5,711 unique *cis*-eGenes and 60,758 unique *cis*-eQTLs, and 145,153 *trans*-eQTL associations, representing 1,382 *trans*-eGenes and 66,847 unique *trans*-eQTLs, under significance thresholds of FDR ≤ 0.05 (corresponding *P* < 1 × 10^−3^) and *P* < 1 × 10^−8^ for *cis*- and *trans*-eQTL associations, respectively. Seventy-five percent of *trans*-eQTLs were also identified as *cis*-eQTLs, which is similar to previous findings (Pierce et al., 2014; Yao et al., 2017). To detect the mediation effects, 999,725 candidate trios (*L*,*C*,*T*) representing 6,217 unique gene pairs (*C*,*T*) were derived from significant *cis*- and *trans*-eQTL associations. For multiple correlated variants linked to each gene pair, we used permutation schemes introduced in *Material and Methods* to control for multiple testing, and for genome-wide unique gene pairs, we used a FDR procedure to control for multiple testing.

### Performance With Adaptive Permutation Scheme

**Figure 1:**
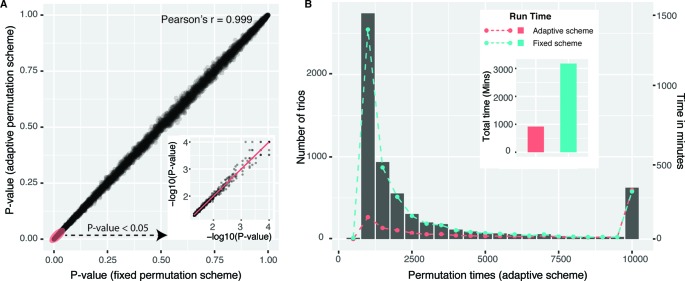
Performance of mediation analysis with adaptive permutation scheme versus fixed permutation scheme. **(A)** Empirical *P* values of 6,217 (*L*,*C*,*T*) trios derived from adaptive scheme (*y*-axis) and fixed scheme (*x*-axis) were shown in Panel A, and the portion of *P*_fixed_ < 0.05 was enlarged in −*log*_10_ scale. **(B)** Trios were grouped by permutation times (in adaptive scheme) and were shown in histogram (*left-side y*-axis). Running time of each group (*right-side y*-axis) using two permutation schemes was overlaid on the histogram with two *colored dash lines*, and the total running time was also shown in the *floating colored bar plot*. To be noted, all trios were executed 10,000 times of permutations in the fixed permutation scheme.

We first compared adaptive permutation scheme implemented in our package with fixed permutation strategy which was commonly adopted by traditional methods, including GMAC (Yang et al., 2017). For each unique gene pair (*C*,*T*) from candidate trios, we selected the most significant *cis*-eQTL for *cis*-eGene *C*, resulting in 6,217 trios. Mediation analyses with fixed permutation scheme (with *N* = 10,000) and adaptive permutation scheme (with *N* = 10,000, *α* = 0.05) were both performed on those 6,217 trios. Empirical *P* values *P*_fixed_ and *P*_adaptive_ were shown in **Figure 1A**, with Pearson’s correlation *r* = 0.999, indicating the two schemes have similar precision. While fixed scheme always executed 10,000 times of permutations for each tested trio, adaptive scheme significantly reduced the permutation times, as shown in the histogram in **Figure 1B**. For example, 68% trios executed less than 2,000 times of permutations. The total time used with adaptive scheme is less than one-third of that with fixed permutation strategy (floating bar plot in **Figure 1B**).

### More Accurate *P* Values and Fewer Permutations with GPD Approximation

**Figure 2:**
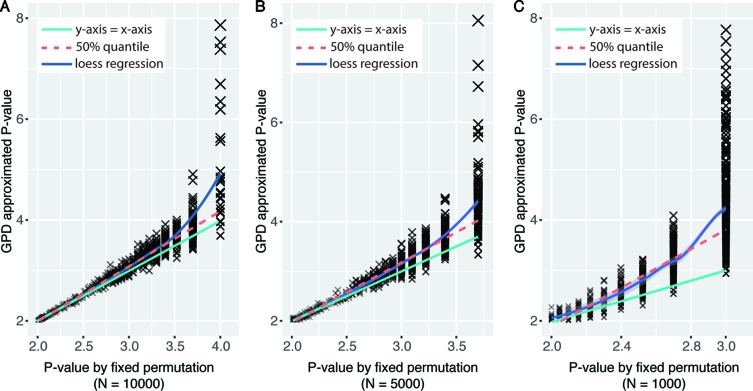
Significance level in mediation analysis estimated under fixed permutation schemes with or without GPD approximation strategy. *X*-axis represents *P* values derived by different fixed permutation schemes (*N* = 10,000, 5,000, and 1,000, respectively) without GPD approximation. *Y*-axis represents *P* values derived with GPD approximation under certain fixed permutation scheme. *P* values were −log10-transformed.

Using generalized Pareto distribution to model the tail of null distribution of permutation statistics could derive more precise empirical *P* values with fewer number of permutations compared with traditional fixed permutation strategy (Knijnenburg et al., 2009). To test the performance of the GPD approximation method implemented in eQTLMAPT, we first randomly selected 1,000 (*L*,*C*,*T*) trios with fixed permutation *P* values were less than or equal to 0.01 (*N* = 10,000). And then we rerun mediation analyses for those trios with GPD approximation under fixed permutation schemes with *N* = 1,000, 5,000, and 10,000. The reason that we only select trios with *P* ≤ 0.01 is because only permutation *P* values at the tail of null distribution can be estimated by the GPD approximation method (see *Model the Tail of the Null Distribution Using GPD*). **Figures 2A**–**C** show the GPD estimated *P* values versus *P* values derived from the fixed permutation scheme (*N* = 10,000, 5,000, and 1,000, respectively), and we can see that GPD-estimated *P* values have higher resolution than fixed permutation scheme. For instance, GPD-estimated *P* values range from 10^−2^ to 10^−8^, while fixed permutation-derived mediation *P* values range from 10^−2^ to 10^−3^, when *N* is set to 1,000. And GPD-estimated *P* values are much smaller than fixed permutation-derived *P* values, which demonstrates that the GPD approximation method has the ability to detect mediation effect more accurately with higher significance resolution.

**Figure 3:**
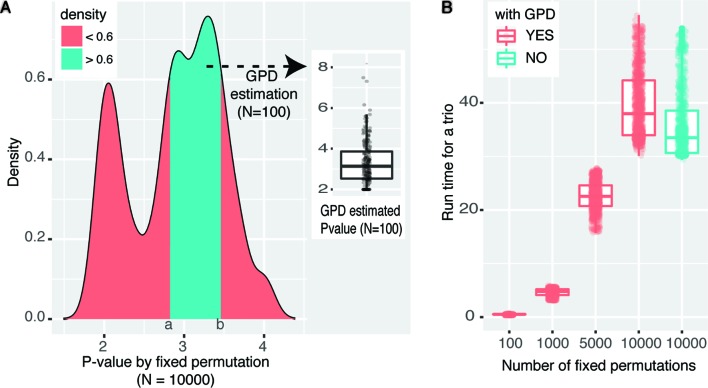
Performance of eQTL mediation analysis with GPD approximation. **(A)** Density plot reflecting the distribution of empirical *P* values under fixed permutation scheme (*N* = 10,000) of 1,000 selected trios with *P*_fixed_ = 0.01 when *N* = 100. The *cyan area* was selected based on the density >0.6, and fixed permutation *P* values were around 10^−3^, when *N* = 10,000. For trios covered by the *cyan area*, GPD-estimated *P* values (*N* = 100) were shown in the *floating boxplot*. **(B)** Time cost for analyzing the same set of trios under various permutation schemes. The *color legend* represents whether GPD estimation process is used. *P* values were −log10-transformed.

To prove the accuracy of the GPD approximation strategy, we first sampled 1,000 trios with *P* value *equal to* 0.01 under the fixed permutation scheme with *N* = 100. It is reasonable to suppose that the significance is likely to be underestimated because of the small *N* (*P*_fixed_ ≤ 0.01). Then we rerun the mediation analyses for those 1,000 trios with *N* set to 10,000, where *P*_fixed_ ≤ 10^−4^. The density plot of *P* values of those 1,000 trios derived under the fixed permutation scheme (*N* = 10,000) was shown in **Figure 3A**, where two peaks around 10^−2^ and 10^−3^ were shown. The peak around 10^−2^ indicates some trios have true significance level around 10^−2^. However, the larger peak centers around 10^−3^ indicate that the significance of a large number of tests is underestimated when *N* = 100. Then we asked whether using GPD approximation strategy can derive *P* values proxy for true *P* values even when *N* was still set to 100. We extracted trios with significance levels between (*a*,*b*) interval (shown in **Figure 3A**) and rerun mediation analyses with GPD approximation and *N* was still set to 100. The distribution of the GPD approximation-derived *P* values was shown as the boxplot in **Figure 3A**, which were centered around 10^−3^, as expected.

The other advantage of using GPD approximation in mediation effect analysis is that with fewer permutations large amount of time cost can be avoided. To achieve a resolution of *P* ≤ 10^−8^, at least 10^8^ permutations should be performed under fixed permutation scheme, while the same resolution could be achieved with only 10^3^ permutations with GPD estimation (see **Figure 2**). **Figure 3B** intuitively shows the time cost for analyzing the mediation effect of a trio under different permutation schemes. One hundred, 1,000, 5,000, and 10,000 permutations were performed in the mediation analysis of the same collection of trios. We can see that the run time is significantly correlated with permutation times. We also tested the time cost caused by the GPD estimation under 10,000 permutations (the two right-most boxplots in **Figure 3B**). We can see that the GPD estimation process only adds a few time cost burden than without GPD estimation, which shows the number of permutations are the most time-consuming. However, *P* value estimates have larger variance for small *N* and converge to the real *P*_perm_ when *N* is getting larger (Knijnenburg et al., 2009). Experimentally, we recommend users to use *N* ≤ 1,000, and the larger *N* will result in more accurate estimated *P* values. In conclusion, by applying GPD approximation strategy, eQTLMAPT can accurately estimate the significance level with fewer permutation operations, which makes the mediation analysis much more efficient.

### Discover *cis*-Mediators of *trans*-eQTLS Using ROSMAP Dataset

To test the speed and discovery performance, we compared eQTLMAPT, combining adaptive permutation scheme and GPD approximation strategy, with GMAC in the discovery of eQTL mediation effects using ROSMAP dataset. For each unique gene pair, we first selected the best trio showing the strongest mediation effect based on the nominal *P* value, resulting in 6,217 candidate trios. Then, we performed mediation analyses using eQTLMAPT and GMAC separately on those 6,217 candidate trios. Both methods adopt permutation tests to adjust *P* values for each trio, and FDR procedure described by Storey and Tibshirani (ST) (Storey and Tibshirani, 2003) to control for multiple testing of gene pairs. To make the comparison comparable, both methods applied the adaptive confounders selection strategy, taking all of the PCs derived from expression profiles as the selection pool of hidden confounders. And both methods adjusted the same fixed covariates (age, sex, RIN, PMI, and batch). We performed *N* = 10,000 permutations for GMAC and performed *N* = 10,000, 5,000, 1,000, and 500 permutations for eQTLMAPT, respectively. In our program, we set *α* = 0.05 in adaptive permutation scheme.

**Figure 4:**
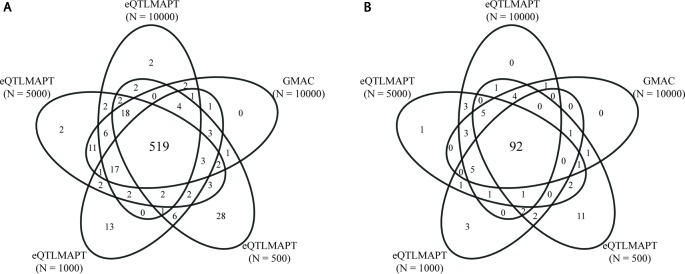
Venn diagram of significant trios at suggestive permutation *P* ≤ 0.05 **(A)** and FDR ≤ 0.25 **(B)** derived by GMAC and eQTLMAPT with different numbers of permutations.

**Table 1** summarizes the performance between eQTLMAPT and GMAC. Both methods detected similar number of trios with suggestive mediation effects (permutation *P* ≤ 0.05) and similar number of significant trios with FDR ≤ 0.25 (Storey and Tibshirani multiple-test controlling method). The Venn diagram in **Figure 4** demonstrated that most significant trios (with suggestive permutation *P* ≤ 0.05 or FDR ≤ 0.25) detected by GMAC can be discovered by eQTLMAPT with *N* = 10,000, 5,000, 1,000, and even 500. For example, among the 113 significant trios with FDR ≤ 0.25 detected by GMAC, 110 (97%) can be discovered by eQTLMAPT with *N* = 10,000, and 104 (92%) can be discovered by eQTLMAPT with *N* = 500. With the similar ability in discovering significant trios, eQTLMAPT is about 90, 40, 8, and 4 times faster than GMAC when *N* = 500, 1,000, 5,000, and 10,000, respectively (**Table 1**). We also noticed that some significant trios detected by eQTLMAPT were missed by GMAC, which might be due to improved *P* value resolution. However, since there is no “true” set of trios with mediation effects, we are not able to compare the true positive rate and false positive rate. In summary, with similar discovery ability, eQTLMAPT is order of magnitudes faster than GMAC. The 519 trios intersected from the five compared strategies with suggestive permutation *P* ≤ 0.05 were available in **Supplementary Table 1**.

**Table 1:** Summary table of performance on speed and discoveries of eQTLMAPT and GMAC.

Software	No. of permutation	No. of trios (adjusted *P* ≤ 0.05)	No. of trios (FDR ≤ 0.25)	Time cost (mins)
GMAC	10,000	578	113	4,438
eQTLMAPT	10,000	580	118	1,131
	5,000	583	115	532
	1,000	577	108	101
	500	596	123	51

### Enrichment Analysis for eQTLs Among GWAS SNPs

**Figure 5:**
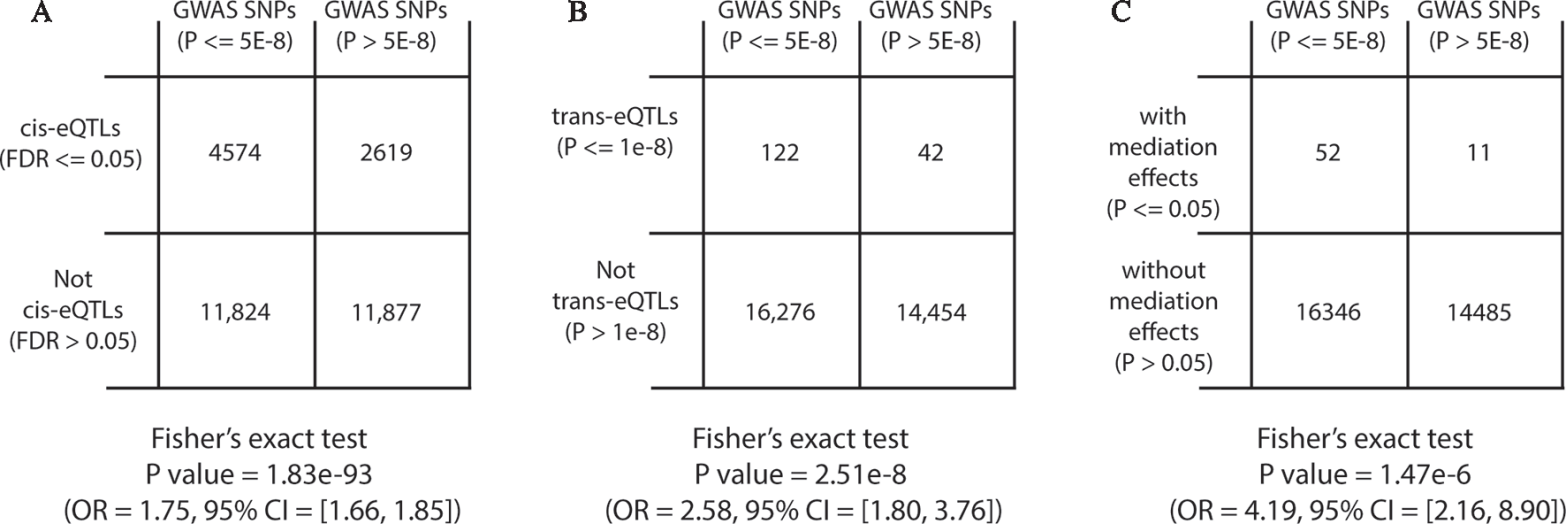
Diagram of two-way contingency tables for Fisher’s exact tests.

We first performed GWAS enrichment analyses for genome-wide significant *cis*-eQTLs (FDR ≤ 0.05) and *trans*-eQTLs (*P* ≤ 1 × 10^−8^). From the NHGRI GWAS catalog (July 2019), 70,971 unique SNPs, reportedly associated with traits and genotyped in ROSMAP dataset, were downloaded (Welter et al., 2013). After pruning correlated SNPs in LD (*r* ^2^ > 0.3) using PLINK and ROSMAP genotype data, 30,894 independent trait-associated SNPs were left, of which, 16,398 SNPs had GWAS *P* ≤ 5 × 10^−8^ and 14,496 SNPs had GWAS *P* ≤ 5 × 10^−8^, respectively. Among SNPs with GWAS *P* ≤ 5 × 10^−8^, 28% were *cis*-eQTLs compared with 18% in SNPs with GWAS *P* ≤ 5 × 10^−8^ (Fisher’s exact test OR = 1.75, with 95% CI = 1.66–1.85 and *P* = 1.83 × 10^−93^; **Figure 5A**). To be noted, the GWAS enrichment method was the same as described in previous work (Westra et al., 2013). In addition, we also observed GWAS enrichment for *trans*-eQTLs (Fisher’s exact test OR = 2.58, with 95% CI = 1.8–3.76, and *P* < 2.51 × 10^−8^; **Figure 5B**). This demonstrated that SNPs known to be associated with traits were more likely to be *cis*/*trans*-eQTLs, which was consistent with previous findings (Fehrmann et al., 2011; Pierce et al., 2014).

Next, we performed GWAS enrichment analysis for eQTLs with significant mediation effects. Among the 999,725 candidate trios, 67,906 trios, representing 27,100 unique SNPs, showed suggestive mediation effects with permutation *P* ≤ 0.05 under fixed permutation scheme (*N* = 10,000). Using the same GWAS enrichment method, we found GWAS SNPs were more likely to have mediation effects (Fisher’s exact test OR = 4.19, with 95% CI = 2.16–8.9, and *P* = 1.47 × 10^−6^; **Figure 5C**), indicating that mediation analysis can help to explain GWAS findings.

### Transcription Factors May Act as *cis*-Mediators

The 519 trios with suggestive permutation *P* ≤ 0.05 (**Supplementary Table 1**) represent 351 unique *cis*-mediators (*cis*-eGenes). Among those *cis*-mediators, we found 14 are TFs, including ZNF488, ZSCAN26, ZNF254, TBX1, FOXS1, ZFP57, ZNF568, ZNF260, ZNF14, GTF2I, ZFX, CSDC2, GTF2IRD2B, and GTF2IRD2. For example, we observed the trio (rs77969091, TBX1, MSC), where TBX1 is the *cis*-eGene and MSC is the *trans*-eGene, and MSC has been predicted to be the target of the transcription factor TBX1 in brain tissue and central nervous system (Marbach et al., 2016). This indicates that *trans*-eQTLs can exert their effects on distant target genes through affecting TFs which act as mediators. However, we did not observe overrepresentation of TFs among *cis*-mediators (Fisher’s exact test *P* = 0.15, compared with 1,665 TFs downloaded from HumanTFDB) (Hu et al., 2018).

## Discussion

There has been intense efforts to identify causal genes and other biomarkers such as RNA, protein, and microbiota underlying complex diseases (Cheng and Hu, 2018; Cheng et al., 2019). One of these efforts is to discover genes regulated by GWAS variants through eQTL analysis. However, less is known regarding how *trans*-eQTLs work on distant genes. The eQTL mediation analysis is a promising tool to uncover the mechanisms underlying *trans*-eQTLs. In order to discover the eQTL mediation effects in whole genome, millions of candidate associations of (eQTL, *cis*-eGene, *trans*-eGene) trios need to be tested, which requires the computational methods to control for multiple testing appropriately. In practice, there are hundreds of variants on average associated with eGenes in both *cis*- and *trans*-manner, which result in huge numbers of candidate trios. For example, in the ROSMAP dataset, nearly 1 million candidate trios need to be tested, which only represent 6,217 unique (*cis*-eGene, *trans*-eGene) pairs. To determine the genome-wide significance of a nominal testing statistics, we need to account for two multiple-testing levels: multiple genetic variants are tested per (*cis*-eGene, *trans*-eGene) pair, and multiple (*cis*-eGene, *trans*-eGene) pairs are tested genome-wide. We used permutation test to correct for the former and FDR estimation to control for the latter.

The traditional permutation scheme, which runs a fixed number of permutations, has to balance the time cost and the *P* value resolution, which is limited by a lower bound. And there is no efficient built-in permutation scheme in current tools aiming at analyzing eQTL mediation effect. To fill this gap, we present eQTLMAPT, which implements a fast and accurate eQTL analysis method with efficient permutation procedures to control for multiple testing. eQTLMAPT can correct for the multiple correlated variants tested *via* three different permutation schemes: the fixed permutation scheme, the adaptive permutation scheme, and the generalized Pareto distribution (GPD) approximation, which models the null distribution of no mediation effects using GPD trained from a few permutation statistics and could accurately estimate the adjusted *P* values without the limitation of lower bound. These strategies implemented in eQTLMAPT greatly accelerated the efficiency of multiple test controling in mediation analyses and provided users higher resolution of estimated significance which would help them distinguish the best signals.

In the analyses of the ROSMAP dataset, we detected 519 trios with suggestive mediation effects (permutation *P* ≤ 0.05), representing 351 unique *cis*-eGenes. Among those *cis*-mediators, we found 14 are TFs, including ZNF488, ZSCAN26, ZNF254, TBX1, FOXS1, ZFP57, ZNF568, ZNF260, ZNF14, GTF2I, ZFX, CSDC2, GTF2IRD2B, and GTF2IRD2. This proves that TFs might play a role in the mediation effects. We also tried to replicate these significant trios with mediation effects in the GTEx dataset analyzed by Yang et al. (2017), and 70 trios, identified by gene pairs, can be replicated with mediation *P* ≤ 0.05 in multiple tissues. For example, the gene pair (MZT2A, AC018804.6) was observed with mediation effects in multiple tissues including brain putamen, fibroblast, colon, esophagus, lung, muscle, pancreas, pituitary, skin, thyroid, and vagina. And the significance of the mediation effect can reach 2 × 10^−7^ in GTEx muscle tissue. This might suggest a common *trans*-eQTL regulatory mechanism across tissues.

There are some limitations of our method and discoveries in the ROSMAP dataset. The discovery of *trans*-eQTLs requires a large sample size because of smaller effect size of *trans*-eQTL associations. A small sample size might cause less replicable *trans*-eQTL signals across studies. The effective sample size of the ROSMAP dataset used in the discovery study is relatively small, which might be the reason that some trios were not able to be replicated in the GTEx dataset, whose sample size is also limited. Besides the transcription factors found in the *cis*-mediators, non-coding genes such as long non-coding RNA (lncRNA), microRNA, snRNA, antisense RNA, and pseudogene, were also detected. The top 3 gene classes are protein coding, pseudogene, and lncRNA genes. Although many studies have shown that non-coding RNAs play key roles in the complex regulatory networks in cell system, most of their functions are still missing (Cheng et al., 2018a; Cheng et al., 2018d; Peng et al., 2019b). Further computational methods and biological experiments are still needed to understand these unknown markers, such as using phynotypes, ontologies, deep learning methods, etc. (Cheng et al., 2016; Cheng et al., 2018c; Peng et al., 2019c; Peng et al., 2019d). In addition, since the gene expression is tissue-specific and cell type-specific, the mediation effects found in brain tissue might not show up in other tissues and cell types. Thus, with the development of single-cell RNA sequencing technologies, further studies should put more attention on cell type-specific mediation effects.

In conclusion, we present eQTLMAPT, an R package which aims to perform eQTL mediation analysis with efficient permutation procedures in multiple testing correction (**Supplementary Figure 1**). Experiments demonstrate that our method provides higher resolution in estimated significance and is an order of magnitude faster than the compared methods. Our method will be helpful in identifying mediation effects, which could allow us to better understand the biological mechanisms underlying *trans*-eQTLs and the regulatory network in the cell.

## Data Availability Statement

Genotype and RNA-seq data of ROSMAP study (in control use): Synapse platform (https://www.synapse.org/#!Synapse:syn3219045). Source code and comprehensive documentation of eQTLMAPT are freely available to download at https://github.com/QidiPeng/eQTLMAPT.

## Author Contributions

TW designed the study and co-implemented the R package, analyzed data, and wrote the paper. QP co-implemented the R package, performed dry experiments, and revised the paper. BL XL, and YL revised the paper and provided suggestions. JP and YW supervised the research, provided funding support, and revised the paper.

## Funding

This work has been supported by the National Key Research and Development Program of China (Nos. 2017YFC1201201 and 2017YFC0907503).

## Conflict of Interest

The authors declare that the research was conducted in the absence of any commercial or financial relationships that could be construed as a potential conflict of interest.

## Supplementary Material

The Supplementary Material for this article can be found online at: https://www.frontiersin.org/articles/10.3389/fgene.2019.01309/full#supplementary-material

Supplementary Figure 1 Overview of functions implemented in eQTLMAPT.
