# Supplementary figures and images for: eQTLMAPT: Fast and Accurate eQTL Mediation Analysis With Efficient Permutation Testing Approaches
Source: Front Genet. 2020 Jan 9;10:1309. doi: 10.3389/fgene.2019.01309 (PMC6970436; doi:10.3389/fgene.2019.01309)

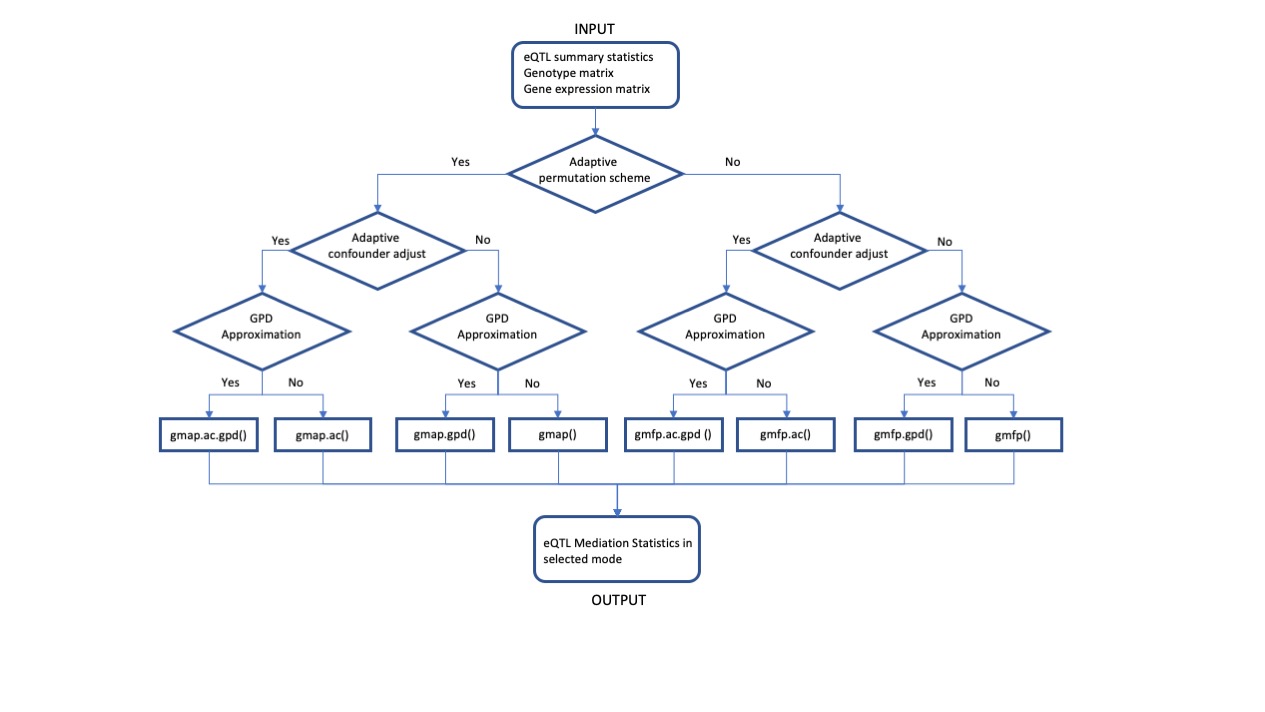

Supplement: Supplementary Figure 1 — Overview of functions implemented in eQTLMAPT. [file Image_1.jpeg]
